# Factors associated with different types of birth attendants for home deliveries: an analysis of the cross-sectional 2010 South Sudan household survey

**DOI:** 10.3402/gha.v9.29693

**Published:** 2016-07-28

**Authors:** Ngatho S. Mugo, Kingsley E. Agho, Anthony B. Zwi, Michael J. Dibley

**Affiliations:** 1 Sydney School of Public Health, The University of Sydney, Sydney, Australia; 2 School of Science and Health, Western Sydney University, Penrith, Australia; 3 School of Social Sciences, University of New South Wales, Kensington, Australia

**Keywords:** skilled birth attendants, maternal health services, home birth, socio-economic factors, South Sudan

## Abstract

**Background:**

In South Sudan, birth deliveries attended by unskilled birth attendants put the mothers and their newborns at increased risk of perinatal morbidity and mortality. The aim of this study was to identify factors associated with delivery by unskilled birth attendants or by unassisted delivery.

**Design:**

We examined data for 2,767 (weighted total) women aged 15–49 years who delivered at home 2 years prior to the South Sudan Household Health Survey 2010. Multinomial logistic regression analyses were used to identify factors associated with delivery by unskilled birth attendants or by unassisted delivery.

**Results:**

The prevalence of delivery by unskilled birth attendants was 19% [95% confidence interval (CI) 17.0, 20.5], by skilled birth attendants (SBAs) was 45% (95% CI 42.4, 47.0), and by unassisted delivery was 36% (95% CI 34.2, 38.6). After adjusting for potential confounders, the following factors were associated with the increased odds for unassisted delivery or delivery by an unskilled birth attendant: mothers with no schooling, who did not attend antenatal care (ANC) during pregnancy, who had lower quality of ANC services, from poor households, or who had no prior knowledge about obstetric danger signs.

**Conclusions:**

We found that non-utilization of maternal health care services, such as ANC, was significantly associated with unattended birth delivery or delivery by unskilled health providers. The increased uptake of SBAs at delivery will require easier access to ANC services, health promotion on the importance and benefits of SBAs for delivery, targeting both mothers and their families, and the training and deployment of more SBAs across the country.

## Introduction

Globally, unassisted delivery or delivery assisted by unskilled birth attendance is a public health concern associated with high maternal mortality and morbidity. According to 2013 estimates, the Sub-Saharan Africa region accounted for 62% (179,000) of global maternal deaths (1). Most of these deaths could have been prevented if mothers had access antenatal care (ANC) during pregnancy, were attended by a skilled birth attendant (SBA) who was able to deal with complications during delivery, and had appropriate care and support in the early postpartum period (2–7). In low and middle-income countries, a significant proportion of women still deliver at home either without or with support from unskilled health providers (8–10).

The dangers of home deliveries include an unhygienic setting, the failure to recognize maternal and fetal distress or complications, the failure to detect maternal and newborn complications during delivery or post-delivery, and inadequate supervision by health care workers (10). Unfortunately, in South Sudan, pregnant women often deliver under such conditions, thus increasing their risks of maternal morbidity and mortality (11).

Access to and use of maternal and child healthcare related services in South Sudan is relatively low. Pregnant women often deliver at home unattended by an SBA due to various socio-economic and physical barriers, such as the costs of health service user fees, lack of transport, inadequate number of skilled health workers, coupled with the insecurity in most of states and the impact of the prolonged civil war, which has led to an almost total destruction of functioning health facilities (11).

In the 2007 South Sudan household survey report, the majority of births (81%) occurred at home with only 11.5% of deliveries at a health facility (12, 13). Of mothers who delivered at home, 30% delivered without assistance, 36% were assisted by relatives or friends, 20% were assisted by traditional birth attendants, and only 10% were assisted by an SBA (12).

In response to Millennium Development Goals (MDGs 5), the government has made efforts to reduce maternal deaths during pregnancy and childbirth and deaths in the first year of life as outlined in the national reproductive health plan (14, 15). The government has also made a recent commitment to the UN Secretary General's Every Woman Every Child initiative with the provision of free reproductive health services and increased access to good quality emergency obstetric care services (16). However, maternal mortality was estimated at 2,054 per 100,000 live births in 2006 and is amongst the highest in the world, highlighting the critical need for developing quality health care during pregnancy, childbirth, and the postpartum period (17).

Several studies have explored the associations between socio-cultural, economic, and other determinants of health that influence women's decisions to deliver at home rather than in a health facility (18–20). However, no study has investigated the factors that influence women choices to deliver at home with unskilled health care providers in South Sudan. Therefore, the aim of this study was to investigate the risk factors associated with delivery in the absence of any assistance and delivery by unskilled birth attendants compared to deliveries attended by an SBA. Our findings will enable policy makers and public health researchers to develop interventions that target vulnerable populations of women and improve access to facility delivery and maternal health services.

## Methods

### Data source

Our dataset was obtained from the South Sudan Household Health Survey second round (SSHHSII) carried out in 2010 that used the Multiple Indicator Cluster Survey (MICS) methodology developed by UNICEF. The SSHHSII is a nationally representative, stratified, cluster sample survey covering the population of South Sudan (13). The aim of the study was to collect health and related indicators to identify the health needs of women and children and to establish priorities for evidence-based planning, decision-making, and reporting. The survey comprised a general questionnaire to collect information on all household members, with three individual questionnaires addressed to specific target groups: women and men aged 15–49 and under-five children. The household and individual questionnaires were used to collect information on demographic characteristics and reproductive history, pregnancy-related information, knowledge and practices concerning family planning, information on child health indicators, and other determinants of maternal health. The dataset comprised information on 9,069 ever-married women, 4,345 men aged 15–49 years and 8,338 under-five children. Details of the SSHHSII sampling methods have been reported (13, 21).

### Sample size determination

The sample size for the SSHHSII was calculated as 10,000 households using the prevalence of under-five child diarrhea as the key indicator assuming a prevalence of 20%, a design effect of 1.5, 16% of the total population to be under-five children, and a participation rate of 90%. The results reported in this paper were based on data from 2,767 women for which the primary outcome was unassisted delivery or delivery by unskilled birth attendants. We estimated that the sample had a 80% power to detect an odds ratio of at least 1.3 or a difference in prevalence of 6.0%, assuming an alpha level of 5%, a prevalence of delivery by unskilled birth attendants or unassisted delivery of 60%, a design effect of 1.25 (based on other surveys) (22), and a total sample of 2,214, which was obtained by dividing 2,767 by the design effect. This provided sufficient statistical power to examine risk factors for the non-use of SBAs that would be of public health significance.

### Study population

**Fig. 1:**
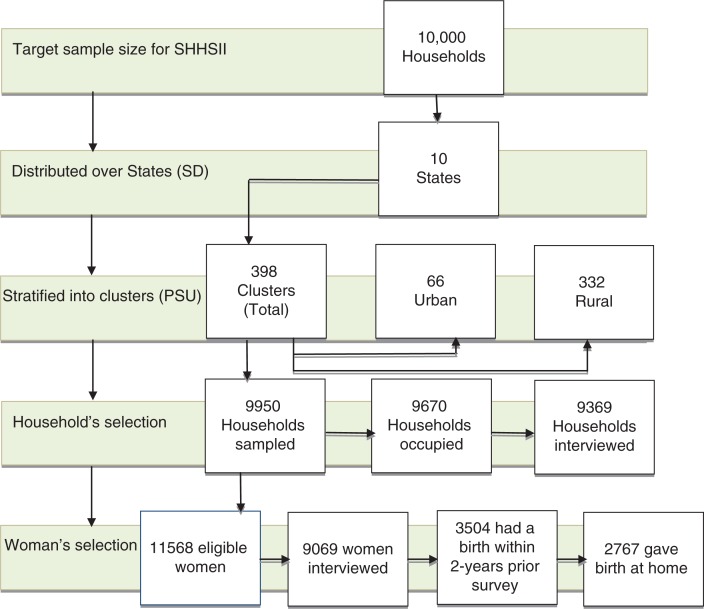
A multiple-stage methodology process for selection of woman at the household level, 2010-SHHSII. SSHHSII: South Sudan Household survey second round. State: is the sampling domain (SD). Cluster: is the primary sampling unit (PSU).

A total of 2,767 of mothers out of 3,504 aged 15–49 years who gave birth within 2 years prior to the survey and delivered their babies at home were included in the analyses. Figure 1 illustrates the selection process for the mother at the household level. Information on delivery assistance was collected from the mothers’ most recent birth. We excluded 457 (14%) of mothers who delivered at health facilities.

### Conceptual framework and study variables

**Fig. 2:**
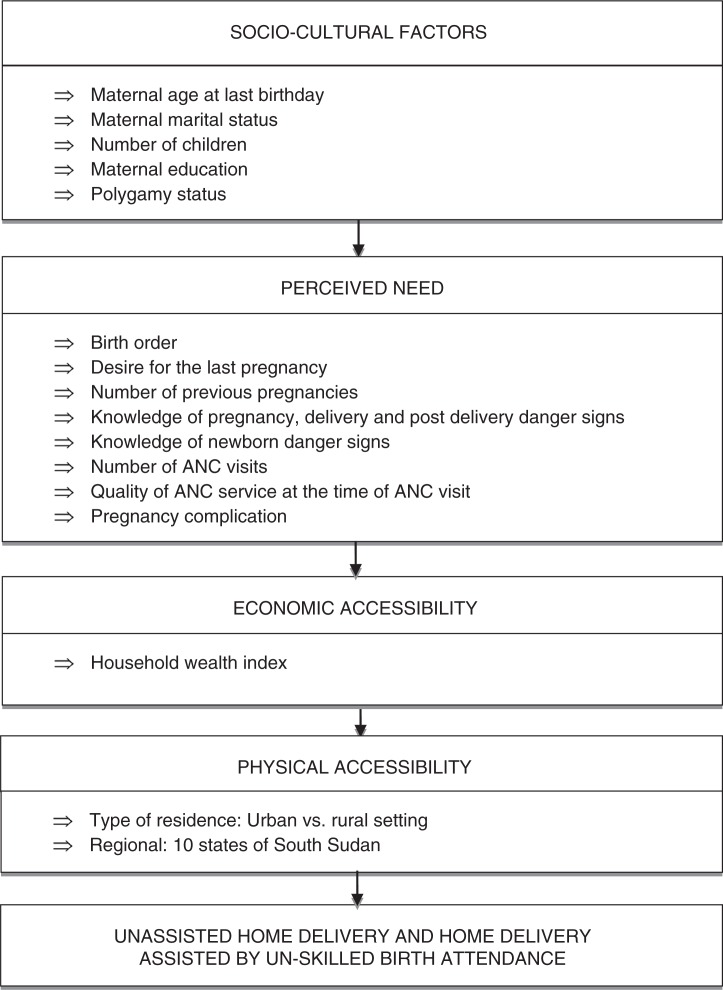
Theoretical framework of risk factors associated with home delivery in South Sudan. This framework was adopted by Gabrysch and Campbell, from the Thaddeus and Maine's three delays model.

We modified the framework by Gabrysch and Campbell (23) to group risk factors that have a potential impact on the access to health care services in developing countries (23, 24). Figure 2 presents the variables included in the analyses. These were selected based on the framework to understand how they were linked to the likelihood of delivery unattended or attended by an unskilled birth attendant. A total of 16 potential risk factors associated with delivery were categorized into 1) socio-cultural factors primarily influencing decisions whether to seek care including maternal age at last birthday, maternal marital status, number of living children, maternal education, and polygamy status; 2) perceived needs and benefits (factors that influence perception of the benefit and need for facility delivery by SBAs including birth order, desire of the last pregnancy, number of previous pregnancy, knowledge of danger signs of pregnancy, delivery and post-delivery, knowledge of newborn danger signs, number of ANC visits, and quality of ANC visits and pregnancy complication); 3) economic accessibility (capacity to cope with costs associated with maternal healthcare including household wealth); and 4) physical accessibility (distance and transport to maternal health services including type of residence and geographical location) (23, 25).

The outcome variable used in analyses was home delivery defined as a childbirth outside a health facility, that is at home or on the way to the health facility, with or without assistance and was classified into 1) mothers who delivered at home unassisted, 2) mothers who delivered at home assisted by an unskilled birth attendant (such as traditional birth attendants, community health workers, and relatives/friends), and 3) mothers who delivered at home assisted by a SBA (such as a doctor, nurse-midwife, village midwife, medical assistance, or health visitor).

Household wealth index was constructed from an inventory of 20 household facilities and assets including material of the dwelling floor, roof, and walls; number of persons per sleeping room; fuel used for cooking; availability of electricity; ownership of radio, television, mobile phone, telephone, refrigerator, and watch; ownership of transport devices such as bicycles, motorcycles/scooters, animal-drawn carts, cars/trucks, and boats; source of drinking water and type of sanitation facility; and ownership of livelihood assets such as animals and land. Details of the wealth index have been reported with a principal components analysis to weight the contribution of items to the index (26). The index was divided into three categories; the bottom one-third of households referred to as poor households, the central one-third as middle level households, and the top one-third as relatively well-off or richer households.

We also generated a variable referred to as quality of ANC services from the ANC services received during pregnancy, which included measuring blood pressure, examining urine and blood samples, administering tetanus toxoid injections, use of iron/folic acid supplements, preventive treatment for malaria, and information on the mode and place of delivery. A woman was considered to have a good quality of ANC service when she received four or more of the above services and to have a lower quality of ANC services when she received fewer than four services (27).

### Ethical approval

The ethics committee of the department of Ministry of Health, Government of South Sudan, approved the survey design of the SSHHSII. All survey respondents provided verbal informed consent; consent for children was obtained through parents, caregivers, or guardians. To maintain participants’ privacy, records were de-identified. The dataset of SSHHSII is not available as a public domain dataset. The first author requested access to the data from Director of Health Social and Demographic Statistics and from the Ministry of Health of South Sudan, and access was granted to use the data for this research.

### Statistical analysis

Analyses were carried out using STATA/MP version 12 (StataCorp, College Station, TX, USA). The ‘Svy’ survey commands were used to allow for adjustments for the cluster-sampling design and sampling weights (28). First, frequency tabulations were conducted with the Taylor series linearization method to estimate confidence intervals (CIs) around prevalence estimates (24). This was followed by contingency table analyses to examine the impact of potential predictors on unassisted delivery or delivery by unskilled birth attendants. Because the outcome variable had three categories, univariable multinomial logistic regressions were conducted to determine the unadjusted odd ratios for unassisted delivery or delivery by unskilled birth attendants. This was followed by multivariable multinomial logistic regression analysis as a four-stage model guided by the conceptual framework to determine the adjusted odds ratios for factors associated with unassisted delivery or delivery by unskilled birth attendants (29).

In the first stage, socio-cultural factors were entered and a manual backward elimination method was used to determine risk factors. In the second stage, the significant factors in the first stage were added to perceived need factors and this was followed by a backward elimination procedure. A similar approach was used for economic accessibility factors and physical accessibility factors in the third and fourth stages. In the final model, the knowledge of newborn and obstetrical danger signs appeared collinear because their univariable and multivariable odds ratios were different; this was explored using chi-square tests. From the multivariable model, odds ratios with 95% CI were used to assess the adjusted independent risk factors associated with unassisted or assisted delivery.

## Results

### Main findings

A total of 3,504 women age 15–49 years gave birth within 2 years prior to the survey of whom 2,767 delivered their babies at home. Of the home deliveries, 19% (95% CI 17.0, 20.5) were unassisted, 45% (95% CI 42.4, 47.0) were assisted by an unskilled birth attendant and only 36% (95% CI 34.2, 38.6) were assisted by an SBA.

### Characteristics of the study population

Table 1 shows the demographics of the study population, which was mainly young married women giving birth for the first time in a monogamous relationship. The women were mostly from rural areas, uneducated, and lacked knowledge about newborn and obstetric danger signs and symptoms. Almost three-fifths of women had not had an ANC visit and three-quarters had a lower quality of ANC services. About one-third lived in poor households and four-fifths had desired their pregnancy. Across the states, the distribution of the study population varied from 8% in Unity to 13% in Upper Nile state.

**Table 1:** Characteristics of the study population according to socio-cultural factors, perceived need, economic accessibility, and physical accessibility, South Sudan household survey second round 2010 (*n*=2,767)

Variable	*N*	%
*Socio-cultural factors*		
Maternal age at last birthday		
15–19 years	212	7.7
20–34 years	1,993	72.0
35–49 years	562	20.3
Maternal marital status		
Currently married	2,137	77.2
Formerly married	454	16.4
Never married (single)	176	6.3
Number of children		
1–2 children	1,345	48.6
3–4 children	828	29.9
5 or more children	593	21.4
Maternal education (*n*=2,766)		
Primary+education	511	18.5
No education	2,255	81.5
Polygamy status (*n*=2,527)		
Husband has one wife	1,500	59.4
Husband has more than one wife	1,027	40.6
*Perceived need*		
Birth order (*n*=2,428)		
First birth	1,158	47.7
Second birth	773	31.9
Third or more birth	497	20.5
Desire for last pregnancy (*n*=2,713)		
Wanted to get pregnant	2,395	88.3
Wanted to get pregnant later/never wanted to get pregnant	318	11.7
Number of previous pregnancies		
1 pregnancy	2,643	95.5
2 or more pregnancies	124	4.5
Knowledge of obstetric danger signs during pregnancy, delivery and post delivery		
Adequate (correct answers for 5 or more questions)	469	17.0
Inadequate (correct answers for 1–4 questions)	2,160	78.1
None (all answers incorrect)	138	5.0
Knowledge on newborn danger signs		
Adequate (correct answers for 5 or more questions)	505	18.3
Inadequate (correct answers for between 1 and 4 questions)	2,133	77.1
None (all answers incorrect)	129	4.6
Number of ANC visits		
4 or more visits	429	15.5
1–3 visits	710	25.7
No visits	1,628	58.8
Quality of ANC service at the time of ANC visits		
Good quality ANC (receiving more than 4 ANC services)	729	26.4
Lower quality ANC (receiving fewer than 4 ANC services)	2,037	73.6
Pregnancy complications^a^		
Yes with 1–2 complications	1,046	37.8
Yes with 3 and more complications	960	34.7
No without complications	760	27.5
*Economic accessibility*		
Household wealth index		
Rich	930	33.6
Middle	921	33.3
Poor	916	33.1
*Physical accessibility*		
Type of resident (total)		
Urban	579	20.9
Rural	2,188	79.1
Geographic location (state)		
Central Equatoria	270.3	9.8
Western Equatoria	288.4	10.4
Eastern Equatoria	236.5	8.5
Lakes	296.1	10.7
Western Bahr el Ghazal	252.5	9.1
Northern Bahr el Ghazal	334.3	12.1
Warap	231.6	8.4
Unity	218.1	7.9
Jounglei	270.1	9.8
Upper Nile	368.9	13.3

a Pregnancy complications included excessive vaginal bleeding, high blood pressure, convulsions, high fever, painful urination, abdominal/back pain, foul-smelling vaginal discharge, and jaundice.

### Risks factors associated with home delivery

Table 2 shows the prevalence of delivery by type of birth attendant. The prevalence of delivery with an SBA was significantly higher for mothers with at least primary education, with a greater knowledge of obstetric and newborn danger signs, with four or more ANC visits, and with higher quality ANC. The prevalence rates for number of previous pregnancies, number of children, polygamy status, desire for pregnancy, and urban/rural residence were similar across the type of birth attendant.

**Table 2:** The prevalence for factors associated with unattended home delivery and home delivery attended by unskilled birth attendants and SBAs according to socio-cultural factors, perceived need, economic accessibility, and physical accessibility from the South Sudan household survey, 2010 (*n*=2,767)

	Unattended home birth		Home birth attended by unskilled health provider		Home birth attended by skilled health provider		
				
Variables	%	95% CI	%	95% CI	%	95% CI	*p*
*Socio-cultural factors*							
Maternal age at her last birthday (years)							
15–19 years	7.0	4.8, 10.0	7.7	6.0, 9.9	8.0	6.0, 9.9	
20–34years	73.2	68.6, 77.4	70.2	66.9, 73.2	73.6	70.4, 76.5	
35–49 years	19.8	16.1, 24.0	22.1	19.5, 24.0	18.4	15.9, 21.1	0.55
Maternal marital status							
Currently married	78.6	73.9, 82.7	73.9	70.6, 76.9	80.7	77.5, 83.5	
Formerly married	16.8	13.0, 21.5	17.6	15.1, 20.4	14.7	12.1, 17.7	
Never married (single)	4.6	3.1, 6.8	8.5	6.7, 10.8	4.6	3.3, 6.3	0.02
Number of children							
1–2 children	47.0	42.1, 51.9	48.2	44.7, 51.9	50.2	44.0, 51.0	
3–4 children	34.8	30.3, 39.7	28.9	25.8, 32.3	28.7	31.7, 38.4	
5 children and more	18.2	14.6, 22.9	22.8	20.0, 25.9	21.1	14.9, 20.5	0.04
Maternal education (*n*=2,766)							
Primary+education	13.4	10.1, 17.6	14.5	12.3, 17.1	25.9	22.7, 29.3	
No education	86.6	82.4, 89.9	85.5	82.9, 87.7	74.0	70.5, 77.2	<0.0001
Polygamy status (*n*=2,527)							
Husband has one wife	55.0	49.9, 59.9	52.5	49.1, 55.9	56.0	52.4, 59.5	
Husband has more than one wife	39.7	34.9, 44.7	37.1	34.0, 40.3	35.8	32.7, 39.1	0.10
*Perceived need*							
Birth order (*n*=2,428)							
First birth	42.0	37.2, 47.1	45.2	41.9, 48.5	37.5	34.3, 40.7	
Second birth	25.9	21.7, 30.6	27.0	24.3, 29.8	30.4	27.4, 33.6	
Third or more birth	17.4	13.9, 21.5	17.0	14.8, 19.5	19.3	16.7, 22.3	0.049
Desire for last pregnancy (*n*=2,713)							
Wanted to get pregnant then	83.3	79.8, 87.0	89.6	87.3, 91.6	84.5	81.9, 86.8	
Wanted to get pregnant later/never wanted to get pregnant	14.8	11.3, 19.1	8.9	7.1, 11.2	12.9	10.8, 15.2	0.03
Number of previous pregnancies							
1 pregnancy	94.2	91.3, 96.2	97.1	96.0, 97.9	94.2	92.1, 95.8	
2 or more pregnancies	5.8	3.8, 8.7	2.9	2.1, 4.0	5.8	4.2, 7.9	0.02
Knowledge of obstetric danger signs during pregnancy, delivery and post delivery							
Adequate for (correct answer 5 or more)	13.8	10.7, 17.7	15.4	13.1, 17.9	20.6	17.6, 24.0	
Inadequate for (correct answer between 1 and 4)	76.9	72.1, 81.1	79.6	76.7, 82.2	76.9	73.5, 80.0	
None for (all incorrect answer)	9.3	6.5, 13.1	5.1	3.8, 6.7	2.5	1.7, 3.7	<0.0001
Knowledge on newborn danger signs							
Adequate for (correct answer 5 or more)	18.4	14.6, 22.9	14.3	12.3, 16.7	23.1	20.0, 26.5	
Inadequate for (correct answer between 1 and 4)	74.2	69.2, 78.5	81.5	78.9, 83.9	73.2	69.7, 76.4	
None for (all incorrect answer)	7.5	5.1, 10.8	4.1	2.9, 5.9	3.7	2.6, 5.3	<0.0001
Number of ANC visit							
4 or more visits	8.7	6.1, 12.1	11.2	9.3, 13.5	24.2	21.1, 27.6	
1–3 visits	19.6	15.9, 23.9	20.6	18.0, 23.5	35.0	31.6, 38.6	
No visits	71.8	66.9, 76.2	68.1	64.8, 71.3	40.7	37.3, 44.3	<0.0001
Quality of ANC service at the time of ANC visits							
Good quality ANC (receiving>4 ANC services)	20.1	16.2, 24.6	16.7	14.3, 19.3	41.6	38.1, 45.1	
Lower quality ANC (receiving<4 ANC services)	79.9	75.4, 83.8	83.3	80.7, 85.7	58.4	54.9, 61.9	<0.0001
Pregnancy complications^a^							
Yes, with 1–2 complications	41.6	36.8, 46.6	35.5	32.3, 38.7	38.6	35.2, 42.1	
Yes, with 3 and more complications	29.0	24.5, 33.9	34.2	31.2, 37.4	38.4	35.0, 42.0	
No, without complications	29.4	24.9, 34.2	30.3	27.4, 33.5	23.0	20.2, 26.0	0.002
*Economic accessibility*							
Household wealth index							
Rich	31.6	27.0, 36.5	31.4	28.2, 34.7	37.4	34.1, 41.0	
Middle	35.5	30.8, 40.6	32.3	29.2, 35.6	33.2	29.9, 36.6	
Poor	32.9	28.1, 38.0	36.3	33.0, 39.7	29.4	26.2, 32.7	0.04
*Physical accessibility*							
Type of resident (total)							
Urban	19.9	16.1, 24.4	18.6	15.9, 21.5	24.3	21.0, 27.8	0.04
Rural	80.1	75.6, 83.9	81.4	78.5, 84.1	75.7	72.2, 79.0	
Geographic location (state)							
Central Equatoria	12.6	9.2, 17.0	8.7	6.7, 11.2	9.5	7.1, 12.6	0.77
Western Equatoria	10.1	7.2, 14.1	9.4	7.2, 12.3	11.9	9.2, 15.3	
Eastern Equatoria	9.6	6.7, 13.6	7.9	5.9, 10.7	8.8	6.6, 11.7	
Lakes	10.4	7.6, 14.0	11.3	9.1, 13.9	10.2	7.9, 13.2	
Western Bahr el Ghazal	8.5	6.0, 11.9	8.8	7.0, 10.9	10.0	7.8, 12.7	
Northern Bahr el Ghazal	10.1	7.3, 13.9	12.8	10.1, 16.3	12.1	9.6, 15.0	
Warap	7.5	5.2, 10.7	9.5	7.6, 11.9	7.5	5.6, 9.8	
Unity	7.3	5.1, 10.3	9.2	7.3, 11.5	6.7	5.0, 8.8	
Jounglei	10.4	7.5, 14.2	9.9	7.7, 12.6	9.3	6.9, 12.4	
Upper Nile	13.5	9.6, 18.6	12.5	10.0, 15.5	14.2	11.1, 17.9	

a Pregnancy complications include excessive vaginal bleeding, high blood pressure, convulsions, high fever, painful urination, abdominal/back pain, foul-smelling vaginal discharge, and jaundice.

Table 3 shows the unadjusted odds ratios from the univariable and multivariable analyses, which indicated that a lower level of education, fewer ANC visits, and poorer quality ANC were risk factors for unassisted delivery and delivery by an unskilled birth attendant. In addition, lacking knowledge about obstetric danger signs, lacking experience with pregnancy complications, being a never-married single mother, and being from a poor household were also significant risk factors for unassisted delivery and the use of unskilled birth attendants.

**Table 3:** Unadjusted and adjusted odds ratios for factors associated with unassisted home delivery and home delivery assisted by unskilled health provider compared to deliveries assisted by SBAs according to socio-cultural factors, perceived need, economic accessibility, and physical accessibility, South Sudan household survey, 2010 (*n*=2,767)

	Unattended home birth						Home birth attended by unskilled health provider					
		
	Unadjusted odd ratios			Adjusted odd ratios^a^			Unadjusted odd ratios			Adjusted odd ratios^a^		
				
Variables	OR	95% CI	P	AOR	95% CI	P	OR	95% CI	P	AOR	95% CI	P
*Socio-cultural factors*												
Maternal age at her last birthday (years)												
15–19 years	1.00						1.00					
20–34 years	1.14	0.71, 1.84	0.58				1.00	0.69, 1.44	0.98			
35–49 years	1.24	0.73, 2.09	0.43				1.26	0.84, 1.88	0.27			
Maternal marital status												
Currently married	1.00			1.00			1.00			1.00		
Formerly married	1.17	0.81, 1.70	0.40	1.00	0.68, 1.48	0.99	1.31	0.99, 1.72	0.06	1.20	0.88, 1.63	0.25
Never married (single)	1.02	0.60, 1.75	0.94	0.89	0.48, 1.66	0.71	2.02	1.32, 3.09	0.001	1.73	1.11, 2.70	0.02
Number of children												
1–2 children	1.00						1.00					
3–4 children	1.29	0.99, 1.70	0.06				1.05	0.84, 1.31	0.69			
5 children and more	0.92	0.66, 1.28	0.62				1.12	0.88, 1.44	0.35			
Maternal education (*n*=2,766)												
Primary or more education	1.00			1.00			1.00			1.00		
No education	2.26	1.58, 3.24	<0.0001	1.65	1.08, 2.51	0.02	2.06	1.58, 2.68	<0.0001	1.43	1.07, 1.91	0.02
Polygamy status (*n*=2,527)												
Husband has one wife	1.00						1.00					
Husband has more than one wife	1.13	0.88, 1.46	0.35				1.10	0.90, 1.35	0.34			
*Perceived need*												
Birth order (*n*=2,428)												
First birth	1.00			1.00			1.00			1.00		
Second birth	0.76	0.56, 1.03	0.07	0.80	0.59, 1.09	0.16	0.74	0.59, 0.92	0.01	0.77	0.61, 0.97	0.03
Third or more birth	0.80	0.57, 1.13	0.21	0.81	0.56, 1.15	0.24	0.73	0.56, 0.95	0.02	0.75	0.57, 0.98	0.04
Desire for last pregnancy (*n*=2,713)												
Wanted to get pregnant then	1.00						1.00					
Wanted to get pregnant later/never wanted to get pregnant	1.16	0.81, 1.67	0.41				0.65	0.48, 0.89	0.01			
Number of previous pregnancies												
1 pregnancy	1.00						1.00					
2+ pregnancy	1.01	0.57, 1.76	0.98				0.49	0.30, 0.79	0.004			
Knowledge of obstetric danger signs during pregnancy, delivery and post delivery												
Adequate for (correct answer 5 or more)	1.00			1.00			1.00			1.00		
Inadequate for (correct answer between 1 and 4)	1.49	1.05, 2.12	0.03	1.45	0.97, 2.16	0.07	1.39	1.059, 1.82	0.02	1.32	0.98, 1.77	0.07
None for (all incorrect answer)	5.49	2.92, 10.3	<0.0001	3.67	1.89, 7.13	<0.0001	2.70	1.56, 4.65	<0.0001	2.08	1.17, 3.71	0.01
Knowledge on newborn danger signs												
Adequate for (correct answer 5 or more)	1.00						1.00					
Inadequate for (correct answer between 1 and 4)	1.27	0.92, 1.77	0.152				1.79	1.38, 2.33	<0.0001			
None for (all incorrect answer)	2.53	1.37, 4.69	0.00				1.80	1.01, 3.20	0.045			
Number of ANC visit												
4 or more visits	1.00			1.00			1.00			1.00		
1–3 visits	1.56	0.99, 2.47	0.058	1.33	0.80, 2.20	0.28	1.27	0.94, 1.71	0.12	1.09	0.79, 1.51	0.59
No visits.	4.92	3.21, 7.55	<0.0001	3.87	2.28, 6.58	<0.0001	3.60	2.71, 4.78	<0.0001	2.13	1.49, 3.04	<0.0001
Quality of ANC service at the time of ANC visits												
Good quality ANC (receiving more than 4 ANC services)	1.00			1.00			1.00			1.00		
Lower quality ANC (receiving fewer than 4 ANC services)	2.83	2.10, 3.80	<0.0001	1.17	0.77, 1.76	0.46	3.55	2.82, 4.48	<0.0001	2.04	1.51, 2.75	<0.0001
Pregnancy complications^b^												
Yes, with 1–2 complications	1.00			1.00			1.00			1.00		
Yes, with 3 and more complications	0.70	0.53, 0.93	0.013	0.65	0.47, 0.90	0.01	0.97	0.77, 1.22	0.79	0.86	0.67, 1.11	0.25
No, without complications	1.19	0.89, 1.59	0.25	0.95	0.68, 1.32	0.76	1.44	1.13, 1.83	0.003	1.11	0.85, 1.45	0.44
*Economic accessibility*												
Household wealth index												
Rich	1.00			1.00			1.00			1.00		
Middle	1.27	0.95, 1.71	0.11	1.16	0.84, 1.61	0.36	1.16	0.92, 1.47	0.20	1.10	0.86, 1.42	0.45
Poor	1.33	0.97, 1.80	0.07	1.27	0.90, 1.80	0.18	1.47	1.16, 1.88	0.002	1.34	1.03, 1.76	0.03
*Physical accessibility*												
Type of resident (total)												
Urban	1.00						1.00					
Rural	1.29	0.94, 1.76	0.11				1.41	1.09, 1.82	0.009			
Geographic location (state)												
Central Equatoria	1.00						1.00					
Western Equatoria	0.64	0.36, 1.134	0.13				0.87	0.55, 1.39	0.56			
Eastern Equatoria	0.82	0.45, 1.47	0.50				0.99	0.59, 1.65	0.96			
Lakes	0.76	0.44, 1.33	0.34				1.20	0.75, 1.94	0.44			
Western Bahr el Ghazal	0.64	0.36, 1.14	0.13				0.96	0.60, 1.56	0.87			
Northern Bahr el Ghazal	0.63	0.37, 1.07	0.09				1.17	0.73, 1.87	0.52			
Warap	0.75	0.42, 1.35	0.34				1.39	0.86, 2.27	0.18			
Unity	0.82	0.46, 1.47	0.51				1.50	0.92, 2.46	0.11			
Jounglei	0.84	0.47, 1.50	0.56				1.16	0.69, 1.96	0.57			
Upper Nile	0.71	0.41, 1.25	0.24				0.97	0.60, 1.55	0.89			

a There are a total number (348) of missing observations, and the odd ratio adjusted for all other variables in the table.

b Pregnancy complications include excessive vaginal bleeding, high blood pressure, convulsions, high fever, painful urination, abdominal/back pain, foul-smelling vaginal discharge, and jaundice.

In the univariable analyses, knowledge of both newborn and obstetrical danger signs were significantly associated with unassisted delivery or delivery with unskilled attendants. However these two variables were collinear (chi-square=1210.0, *p*=<0.0001) and could not both be included in the final multivariable model. Therefore, only knowledge of obstetric danger signs was used.

## Discussion

The results of this study indicate that a high proportion of South Sudanese mothers still deliver at home, often unassisted or assisted by unskilled birth attendants, thus increasing their risk of maternal morbidity. The prolonged civil war may have further contributed to the country having the world's lowest percentage of births attended by SBAs (11). Since the majority of births take place at home, high priority should be given the short- term strategy of making every delivery safe by providing the presence of a trained SBA. In the long term, in order to reduce maternal morbidity and mortality, the focus should be to expand access to improved delivery care in equipped maternity facilities to accommodate the needs of women during pregnancy, delivery, and post-delivery.

The main factors associated with unassisted delivery and delivery assisted by an unskilled attendant were low levels of education, inadequate prior knowledge of pregnancy, lack of knowledge of delivery and post-delivery danger signs, and the failure to use maternity care services. These factors were significantly associated with women's choice to deliver without an SBA.

The failure to use maternal health services, such as ANC visits, was a predictor of women delivering without an SBA. We found that women who had no ANC visits were at an increased risk of delivery with unskilled birth attendants or unassisted delivery, in comparison with women who had at least one to three ANC visits. This finding is similar to that of other studies in Tanzania, Cambodia, Ethiopia, and South Sudan (21, 30–32). ANC visits allow the early detection of obstetrical complications and also give the health provider an opportunity to discuss delivery plans and influence the decision to have an SBA. However, low access to and use of these services may be the result of inadequate or absent services at the local health facility, which may negatively impact mothers seeking medical care from SBAs (21, 32). Hence, there is a need for better trained community-based health staff in order to increase the uptake of delivery by SBAs. The government of South Sudan needs to improve locally available and accessible antenatal, intra-partum, and immediate prenatal care. It is also essential carry out community outreach in order to educate and increase awareness on family planning and the availability and importance of maternal health services.

The quality of services during ANC visits is an important factor that can influence where mothers deliver their babies. We found that mothers who had lower quality ANC services, such as examining their blood pressure and blood and urine sample tests, were at increased risk of unassisted delivery or delivery with an unskilled birth attendant. This finding is consistent with that of studies in Nepal, Ghana, and Congo (33–35). In South Sudan, low quality ANC services are associated with poor health infrastructure, coupled with lack of medical supplies. The supply of medical supplies may be affected not only shortages of medicines but also, in most of the country, the unavailability of refrigeration, which is necessary for the long-term storage of drugs (11, 36). In addition, health providers are insufficiently trained to meet the health needs of mothers and are often confronted with the inadequate and delayed payment of their salaries (11). Therefore, there is a need for the government to improve existing health facilities and ensure the proper training of health providers, including emergency care services. Improving the payment of salaries to health providers across the health sector, as well as providing incentives to health providers in remote and rural areas, would go a long way to increase the rate of delivery of babies at health facilities or at home with assistance from an SBA.

In Ghana and Turkey (37–39), household poverty is strongly associated with unskilled birth attendants, a conclusion that was confirmed in our study, which showed that mothers from poor households had a significantly higher risk of unassisted delivery or delivery by an unskilled birth attendant. In South Sudan, poverty affects most households (17) and most mothers from poor households reside in rural and remote areas and have had no schooling (21). Mothers who live in such conditions are confronted with challenges such as minimal access to health services as a result of distance, lack of transport, or the high cost of transport to services (40). In order to increase access to SBAs for all births, the government needs to implement policies that would ensure that all women, regardless of their ability to pay, have access to appropriate and affordable services for maternal and newborn care (16). Women should also be offered conditional cash transfers to encourage them to deliver by SBA, either at home or in a health facility.

Mothers in South Sudan lack the essential knowledge to take basic preventive measures for their own health during pregnancy and childbirth, and that of their newborns, which is in line with previous studies in Mali and Zambia (41, 42). Inadequate health knowledge was linked to not using ANC services in this study and a previous study in South Sudan (21). The South Sudan government needs to implement innovative strategies to increase awareness and access to reproductive health services, such as access to routine ANC visits, and to identify women early in pregnancy and encourage them to use these services. This could be achieved at the village level through enhanced health promotion and by training community health workers, such as TBAs, to provide education about maternal and child health and services. Encouraging girls to attain at least primary level of education is essential to improve access to ANC services, and knowledge and access to SBAs at the delivery of their children.

### Strengths and limitations

This paper is the first analysis of the potential risk factors associated with unassisted delivery or delivery assisted by unskilled attendants. The important strengths include a high response rate (78%) and an appropriate adjustment in the analyses for the sampling design. Due to the large sample size, we were able to identify a variety of risk factors associated with the delivery method. We collected data from the most recent birth within 2 years of the survey to minimize the potential recall bias of the mothers.

The limitations of this study include the use of cross-sectional survey data that restricted the interpretation of the causality of risk factors associated with home birth. The potential risk factors were restricted to factors available in SSHHSII data and the data relied on the mother's recall of details about her pregnancy and childbirth.

## Conclusions

In this study, births that were either unattended or attended by an unskilled assistant was higher among women who were poorer and more marginalized, and had less access to care during pregnancy. Implementing strategies that target women at the community level, for example, by training and deploying community health workers to identify women who need access to care, could increase the number of women who deliver with SBAs and could help overcome the barriers to accessing this service. The government needs to address the socio-economic factors that prevent women from using maternal health services and provide free reproductive services and conditional cash transfers to encourage women to deliver with SBAs. Also, more attention should be paid to improving infrastructure (such as better access to paved roads, as well as adequate maternal health services and medical supplies) in both rural and remote areas. Training and improving the skills of both TBAs and health staff is essential in order to reduce maternal mortality and morbidity in South Sudan.

## Paper Context

Globally unassisted delivery or delivery assisted by unskilled birth attendants is associated with high maternal mortality and morbidity. In South Sudan, most pregnant women often deliver at home unattended by skilled birth attendants (SBAs), but the various factors leading to these unattended deliveries have not been assessed. Therefore, to fill this knowledge gap, this study investigated the risk factors associated with delivery in the absence of any assistance and delivery by unskilled birth attendants compared with deliveries attended by an SBA.

## References

[1] WHO, UNICEF, UNFPA, The World Bank, The United Nations (2014). Trends in maternal mortality: 1990 to 2013. Estimates by WHO, UNICEF, UNFPA, The World Bank and the United Nations Population Division.

[2] WHO, UNICEF, UNFPA, The World Bank (2012). Trends in maternal mortality: 1990 to 2010.

[3] United Nations (2014). The millennium development goals report.

[4] Lawn JE, Tinker A, Munjanja SP (2006). Where is maternal and child health now?. Lancet.

[5] Ronsmans C, Graham WJ (2006). Maternal survival 1 – maternal mortality: who, when, where, and why. Lancet.

[6] Kinney MV, Kerber KJ, Black RE, Cohen B, Nkrumah F, Coovadia H (2010). Sub-Saharan Africa's mothers, newborns, and children: where and why do they die?. PLoS Med.

[7] UNICEF (2008). Progress for children: a report card on maternal mortality.

[8] Koblinsky M, Matthews Z, Hussein J, Mavalankar D, Mridha MK, Anwar I (2006). Going to scale with professional skilled care. Lancet.

[9] Montagu D, Yamey G, Visconti A, Harding A, Yoong J (2011). Where do poor women in developing countries give birth? A multi-country analysis of demographic and health survey data. PLoS One.

[10] Abebe F, Berhane Y, Girma B (2012). Factors associated with home delivery in Bahirdar, Ethiopia: a case control study. BMC Res Notes.

[11] Ministry of Health, Government of South Sudan (2012). Health sector development plan 2012–2016.

[12] Ministry of Health Government of Southern Sudan (MOH-GOSS), Southern Sudan Commission for Census Statistics and Evaluation (SSCCSE) (2007). Southern Sudan household health survey 2006.

[13] Ministry of Health (MoH), National Bureau of Statistics (NBS) (2013). The republic of South Sudan: the Sudan household health survey 2010.

[14] Government of South Sudan (GoSS), Ministry of Health (MoH), UNFPA (2007). Southern Sudan maternal, neonatal and reproductive health strategy: action plan 2008–11.

[15] Government of Southern Sudan, Ministry of Health (2013). National reproductive health strategic plan 2013–2016.

[16] WHO (2015). Accountability for women's and children's health: South Sudan commitment- every woman every child.

[17] National Bureau of Statistics (NAB) (2012). National baseline household survey 2009 – report for South Sudan.

[18] Choudhury N, Moran AC, Alam MA, Ahsan KZ, Rashid SF, Streatfield PK (2012). Beliefs and practices during pregnancy and childbirth in urban slums of Dhaka, Bangladesh. BMC Public Health.

[19] Pfeiffer C, Mwaipopo R (2013). Delivering at home or in a health facility? Health-seeking behaviour of women and the role of traditional birth attendants in Tanzania. BMC Pregnancy Childbirth.

[20] Baral Y, Lyons K, Skinner J, van Teijligen E (2010). Determinants of skilled birth attendants for delivery in Nepal. Kathmandu Univ Med J.

[21] Mugo NS, Dibley MJ, Agho KE (2015). Prevalence and risk factors for non-use of antenatal care visits: analysis of the 2010 South Sudan household survey. BMC Pregnancy Childbirth.

[22] Uganda Bureau of Statistics (UBOS), Macro International Inc (2006). Uganda Demographic and Health Survey (UDHS) 2006.

[23] 
Gabrysch S, Campbell OMR (2009). Still too far to walk: literature review of the determinants of delivery service use. BMC Pregnancy Childbirth.

[24] Thaddeus S, Maine D (1994). Too far to walk: maternal mortality in context. Soc Sci Med.

[25] Belaid L, Ridde V (2014). Contextual factors as a key to understanding the heterogeneity of effects of a maternal health policy in Burkina Faso?. Health Policy Plan.

[26] Filmer D, Pritchett LH (2001). Estimating wealth effects without expenditure data – or tears: an application to educational enrollments in states of India. Demography.

[27] Lincetto O, Mothebesoane-Anoh S, Gomez P, Munjanja S (2006). Antenatal care. Opportunities for Africans newborns: practical data policy and programmatic support for newborn care in Africa.

[28] StataCorp (2013). Stata: release 13. Statistical Software.

[29] Victora CG, Huttly SR, Fuchs SC, Olinto MT (1997). The role of conceptual frameworks in epidemiological analysis: a hierarchical approach. Int J Epidemiol.

[30] Mpembeni RN, Killewo JZ, Leshabari MT, Massawe SN, Jahn A, Mushi D (2007). Use pattern of maternal health services and determinants of skilled care during delivery in Southern Tanzania: implications for achievement of MDG-5 targets. BMC Pregnancy Childbirth.

[31] Yanagisawa S, Oum S, Wakai S (2006). Determinants of skilled birth attendance in rural Cambodia. Trop Med Int Health.

[32] Mengesha ZB, Biks GA, Ayele TA, Tessema GA, Koye DN (2013). Determinants of skilled attendance for delivery in Northwest Ethiopia: a community based nested case control study. BMC Public Health.

[33] Khanal V, Adhikari M, Karkee R, Gavidia T (2014). Factors associated with the utilisation of postnatal care services among the mothers of Nepal: analysis of Nepal demographic and health survey 2011. BMC Womens Health.

[34] Esena RK, Sappor MM (2013). Factors associated with the utilization of skilled delivery services in the Ga East Municipality of Ghana Part 2: barriers to skilled delivery. Int J Sci Tech Res.

[35] Tchibindat F, Martin-Prevel Y, Kolsteren P, Maire B, Delpeuch F (2004). Bringing together viewpoints of mothers and health workers to enhance monitoring and promotion of growth and development of children: a case study from the Republic of Congo. J Health Popul Nutr.

[36] Mugo N, Zwi AB, Botfield JR, Steiner C (2015). Maternal and child health in South Sudan: priorities for the post-2015 Agenda. Sage Open.

[37] Arthur E (2012). Wealth and antenatal care use: implications for maternal health care utilisation in Ghana. Health Econ Rev.

[38] Abor PA, Abekah-Nkrumah G, Sakyi K, Adjasi CKD, Abor J (2011). The socio-economic determinants of maternal health care utilization in Ghana. Int J Soc Econ.

[39] Celik Y, Hotchkiss DR (2000). The socio-economic determinants of maternal health care utilization in Turkey. Soc Sci Med.

[40] New Sudan Centre for Statistics and Evaluation (NSCSE), UNICEF (2004). Towards a baseline: best estimates of social indicators for Southern Sudan.

[41] Gage AJ (2007). Barriers to the utilization of maternal health care in rural Mali. Soc Sci Med.

[42] Stekelenburg J, Kyanamina S, Mukelabai M, Wolffers I, Roosmalen J (2004). Waiting too long: low use of maternal health services in Kalabo, Zambia. Trop Med Int Health.

